# Predicting alcohol use disorder remission: a longitudinal multimodal multi-featured machine learning approach

**DOI:** 10.1038/s41398-021-01281-2

**Published:** 2021-03-15

**Authors:** Sivan Kinreich, Vivia V. McCutcheon, Fazil Aliev, Jacquelyn L. Meyers, Chella Kamarajan, Ashwini K. Pandey, David B. Chorlian, Jian Zhang, Weipeng Kuang, Gayathri Pandey, Stacey Subbie-Saenz de. Viteri, Meredith W. Francis, Grace Chan, Jessica L. Bourdon, Danielle M. Dick, Andrey P. Anokhin, Lance Bauer, Victor Hesselbrock, Marc A. Schuckit, John I. Nurnberger, Tatiana M. Foroud, Jessica E. Salvatore, Kathleen K. Bucholz, Bernice Porjesz

**Affiliations:** 1 Department of Psychiatry, State University of New York, Downstate Medical Center, Brooklyn, NY USA; 2 Department of Psychiatry, Washington University School of Medicine in St Louis, St Louis, MO USA; 3 Department of Psychiatry, Virginia Commonwealth University, Richmond, VA USA; 4 Faculty of Business, Karabuk University, Karabük, Turkey; 5 Brown School of Social Work / Department of Psychiatry, Washington University in Saint Louis, St. Louis, MO USA; 6 Department of Psychiatry, University of Connecticut School of Medicine, Farmington, CT USA; 7 Department of Psychiatry, University of California, San Diego School of Medicine, La Jolla, CA USA; 8 Departments of Psychiatry and Medical and Molecular Genetics, Indiana University School of Medicine, Indianapolis, IN USA; 9 Department of Medical and Molecular Genetics at Indiana University School of Medicine, Indianapolis, IN USA; 10 Department of Psychology, Virginia Commonwealth University, Richmond, VA USA; 11 Virginia Institute for Psychiatric and Behavioral Genetics, Virginia Commonwealth University, Richmond, VA USA

**Keywords:** Genetics, Predictive markers

## Abstract

Predictive models for recovering from alcohol use disorder (AUD) and identifying related predisposition biomarkers can have a tremendous impact on addiction treatment outcomes and cost reduction. Our sample (*N* = 1376) included individuals of European (EA) and African (AA) ancestry from the Collaborative Study on the Genetics of Alcoholism (COGA) who were initially assessed as having AUD (DSM-5) and reassessed years later as either having AUD or in remission. To predict this difference in AUD recovery status, we analyzed the initial data using multimodal, multi-features machine learning applications including EEG source-level functional brain connectivity, Polygenic Risk Scores (PRS), medications, and demographic information. Sex and ancestry age-matched stratified analyses were performed with supervised linear Support Vector Machine application and were calculated twice, once when the ancestry was defined by self-report and once defined by genetic data. Multifeatured prediction models achieved higher accuracy scores than models based on a single domain and higher scores in male models when the ancestry was based on genetic data. The AA male group model with PRS, EEG functional connectivity, marital and employment status features achieved the highest accuracy of 86.04%. Several discriminative features were identified, including collections of PRS related to neuroticism, depression, aggression, years of education, and alcohol consumption phenotypes. Other discriminated features included being married, employed, medication, lower default mode network and fusiform connectivity, and higher insula connectivity. Results highlight the importance of increasing genetic homogeneity of analyzed groups, identifying sex, and ancestry-specific features to increase prediction scores revealing biomarkers related to AUD remission.

## Introduction

National surveys on alcohol use statistics and Alcohol Use Disorder (AUD) studies show that only one-third of individuals with AUD attempted to quit drinking every year^1^. Of them, only 25% were successful in reducing alcohol consumption for more than a year^1^. At the same time, there are ongoing debates over courts and correctional programs admitting individuals into rehabilitation programs given their efficacy and program’s outcome^2,3^. Therefore, it is of great importance to be able to identify AUD resilience and readiness to recover features including predisposition characteristics that can predict a change in drinking behavior, consequently impacting therapeutic approaches to AUD, helping individuals overcome addiction and overall reducing state, and federal associated financial burden. Few studies have investigated the characteristics of those with AUD who reduce alcohol consumption, but they have often focused on psychosocial aspects^3^ and initial consumption measurements^4,5^, leaving physiological and genetic variables unexplored.

Recently, the ability to use multimodal multi-features machine learning (ML) applications has started to revolutionize biomedical research enabling to classify and predict diseases, as well as a better understanding of development and treatment outcomes, outperforming more classical analyses such as regressions^6–8^. Significant ML Support Vector Machine (SVM) classifier models were found for complex disorders, including schizophrenia, bipolar disorder, and depression, identifying discriminating features^9^. Our own study^8^ and others^7^, have shown that the accuracy of ML models increases by using multimodal, multi-features approaches to describe complex disorders, permitting a variety of measurement domains that could be brought to bear on different aspects of disease pathology^7^. Indeed, ML studies calculating AUD classifiers/predictive models have employed genetic loci^8^, psychosocial^7^, family history^8^, and electrophysiological (EEG) measurements^8^ as features in a multimodal analysis. In the current ML study, we have utilized EEG, genetics, medication intake, and demographic as predisposition characteristics to predict AUD remission. EEG measurements, especially resting-state functional connectivity (EEG-FC) have been shown to be a reliable diagnostic tool and classifier in AUD and other brain disorders such as post-traumatic stress disorder, and bipolar disorder^10^. Polygenic risk scores (PRS), which summarize the effects of genome-wide association study (GWAS) markers to measure the genetic liability to a trait or a disorder, have shown promise in predicting human complex traits and diseases^11,12^. Several GWAS studies tested alcohol-related PRS for association with AUD phenotypes, using PRS related to risky behaviors, alcohol-use problems, and alcohol consumption with encouraging results^13,14^. We also tested demographic features including marital and employment status which have been found to be associated with a reduction in alcohol consumption and remission^1^ from AUD. Current medication intake was added as a potential feature to the calculated AUD remission predictive model. Alcohol misuse targets areas of the brain, altering mental states such as emotion^15^ and cognition^16^, thus affecting an individual’s capability to cope with the challenges involved in the relapse/recovery processes^17^. Medication can restore brain regulation abilities, potentially strengthening and stabilizing individual mental abilities, thus supporting AUD remission. The substantial impact that marital and occupational status has for those with AUD^18,19^, indicates stabilizing and supportive environmental effects.

The present study, therefore, aims to create an ML model, predicting future AUD remission among individuals who had met criteria for active DSM-5 AUD at their first interview but no longer meet criteria for current DSM-5 AUD at their next interview. It should be noted that remission is a complex, multidimensional process; this study focuses on the reduction of alcohol use and of AUD symptoms to subclinical levels, which is one component of remission^20^. We used longitudinal multidimensional data from COGA (e.g., clinical, electrophysiological, GWAS, demographics), including individuals of European Ancestry (EA) and African Ancestry (AA). COGA collects data and follows individuals with AUD, providing a unique opportunity to compare an individual’s AUD status over the development of their addiction and during their remission. Most importantly the diverse COGA data-enabled stratified analyses, increased group homogeneity, creating an individualized model, and discriminative key features for every group. To further increase group ancestry genetic homogeneity we calculated the models when ancestry was based on self-report and again based on genetics calculated with ancestral principal components (PCA)^21^. Our central hypothesis was that model based on multidimensional features will result in a better prediction than singular modality and that being married, employed, and taking medication will predict remission. Using stratification to control for the confounding variables, sex, and ancestry, we expected to find differences in the prediction models between the groups, with higher accuracy scores when the ancestry was calculated using genetic data. We also examined the most discriminative features in the predictive models, enhancing our understanding of neurophysiological, genetic, and socio-demographic characteristics underlying AUD resilience and recovery.

## Materials and methods

### Participants

The data consisted of 1376 participants (826 males and 550 females) from COGA, including EA and AA individuals. Data from seven collection sites were included in this study. The experimental protocols were approved by each site’s institutional review board, and informed consent (for those over 18 years of age) or assent (for those under 18 years of age) was obtained from all participants. Ascertainment and assessment procedures of COGA recruits have been described elsewhere^22^ and in Supplementary Materials. Only individuals who met criteria for lifetime DSM-5 AUD and who participated in at least two interviews were included in the sample for these analyses. We examined only participants who were diagnosed as DSM-5 AUD at their initial laboratory visit and reassessed years later, at a follow-up visit, when they were divided into two groups: (1) continued AUD: met criteria for active DSM-5 AUD at time 1 and time 2, and (2) remitted AUD: no longer met criteria for active DSM-5 AUD at time 2. The AUD and remission phenotypes were based on information from two consecutive interviews. Both continued AUD and remitted AUD met criteria for current AUD at the first interview, defined as the presence of two or more AUD criteria within the previous 12 months. Remission at the second interview was defined as the absence of all AUD criteria other than craving for at least 12 months and either low-risk drinking or abstinence (*n* = 688, 413 males, 275 females, mean age at initial visit: 30.62 ± 9.41, mean number of years between visits = 4.6 ± 1.7). The continued AUD group met criteria for current AUD at both interviews (*n* = 688, 413 males, 275 females, mean age: 30.79 ± 9.36, mean number of years between visits = 4.8 ± 1.6). The analysis was done on the data collected during the first visit to predict remission status at the second visit. In a series of analyses, the groups were further divided according to ancestry (EA, AA) and sex (male, female). Stratified analysis by ancestry was done twice: once with ancestry identified by self-report and once identified by implementing SNPrelate^23^ to estimate principal components from GWAS data which was subsequently used to determine EA and AA. Sex, ancestry, and features’ missing values dictated a series of analyses that included different subsets of subjects. All groups were matched on age. A full description of each of the groups can be found in Supplementary Tables S1–S4.

### Procedure

#### EEG data acquisition and preprocessing

EEG was recorded for 4 min as the participants were sitting on a comfortable chair and were instructed to stay awake with their eyes closed and not to move. Participants sat in a dimly lit, sound-attenuated RF-shielded booth (Industrial Acoustics, Inc., Bronx, NY, USA) with 64-channel electrode cap (Electro-Cap International, Inc., Eaton, OH, USA) based on the extended 10–20 System. EEG recording and preprocessing procedures are described in Supplementary Materials.

### Feature extraction

#### EEG extracted features

A full description of EEG functional connectivity calculation (using MNE package)^24^ can be found in Supplementary Materials and Supplementary Table S5. Briefly, The FreeSurfer parcellation scheme (aparc.lh/rh), based on the Desikan–Killiany Atlas^25^, was used to define 68 cortical regions from both hemispheres (list of ROIs in Supplementary Table S5). We computed spectral coherence^26^ to measure functional connectivity (FC) between EEG signals of 68 regions of interest (ROI) at specific frequency bands: theta (4–8 Hz), alpha (8–12 Hz), beta (12–30 Hz), and gamma (30–60 Hz) with no overlap between frequencies. The following electrophysiological features were extracted: for each of the frequency bands (theta, alpha, beta, and gamma), a 68 × 68 ROIs matrix of coherence was created for each participant resulting in 9221 features. Each of these features represents an EEG coherence functional connectivity (EEG-FC) between two ROIs.

### PRS features

PRS based on GWAS weights from 47 phenotypes were derived from 12 publicly available large-scale GWAS of alcohol-related traits conducted in EA and AA males and females including GWAS of alcohol consumption^27–29^, DSM-IV alcohol dependence^28,30,31^, and a maximum number of alcoholic drinks within 24 h. Additional PRS were derived from GWAS of other traits known to correlate with alcohol use and problems, including educational attainment^32,33^, anxiety disorders^34^, personality traits(e.g., aggression^35^, neuroticism^33,36^), depression^33^, subjective wellbeing^32^, brain structure^37^, and environmental sensitivity^38^ (overall number of PRS features = 1162). Details regarding the discovery of GWAS, including the number of individuals who participated in the GWAS and phenotypes, can be found in Table S6. Information on genotyping and quality control is available in the Supplemental Materials. Briefly, the well-established process of clumping and thresholding was used^39^ where single nucleotide polymorphisms (SNPs) from discovery GWAS were clumped based on linkage disequilibrium (LD) in the 1000 genomes EUR panel using PLINK 1.9^40^, based on an R2 = 0.25, with a 500 kb window. SNPs were weighted using the negative log of the association *p* values. Scores were based on differing thresholds of GWAS *p* values (*p* < 0.0001, *p* < 0.001, *p* < 0.01, *p* < 0.05, *p* < 0.10, *p* < 0.20, *p* < 0.30, *p* < 0.40, *p* < 0.50). PRSs were converted to Z-scores for interpretation.

#### Marital, employment status, and medication intake

This information was gathered as part of COGA’s assessment procedure (see^22^ Supplementary Materials for further description). Assessment about prescribed medication intake during the last 30 days includes medication for sleep, anxiety, headaches, birth control, depression, energy, containing steroids, and another category listed as ‘other medication’.

### Machine learning analysis

Z Normalization was applied to all the features to maintain a common scale, without distorting differences in the ranges of values. Regularization methods were used to control for variables overfitting, enhancing the interpretability, and prediction accuracy of the calculated models. We used the least absolute shrinkage and selection operator (LASSO) penalty approach shown by Tibshirani^41^ for feature selection. The sparsity property of LASSO which generates coefficient estimates of exactly zero, shrinks the estimation variance resulting with a more interpretable model^42^. Previous use of this application for genomic data^43^ has shown that the selective number of discriminating features can reach satisfactory classification. Regularization parameters were determined using a tenfold cross-validation (CV) procedure, with the label: continued AUD vs. remitted AUD as the response variable. The reduced set of the most discriminant features with non-zero coefficient was fed into the model to predict participants status to either continued AUD group or remitted AUD group. A supervised linear-kernel SVM that included parameter optimization was trained with a tenfold CV procedure to classify participants into the two groups. The tenfold CV procedure involved randomly dividing the participants into ten equal groups, training the classifier on nine of them, and tested the trained model on the left out one. To ensure randomization of the participants in the calculated model, the dataset was shuffled before every fold. To take advantage of the randomization procedure, we repeated this process ten times, averaging the output results. CV was applied to all models with additional training/testing (70:30) validation analysis to confirm results in the larger samples (EA male and females). Model performance was evaluated by calculating the number of true positives (TP, number of correctly classified remitted AUD) and true negatives (TN, number of correctly classified continued AUD) scores. We computed the classification accuracy as the ratio of sum of TP and TN divided by the sum of all classified subjects. Area under curve (AUC)^7^ was used to evaluate the classification models. More description of AUC calculation and comparison can be found in Supplementary Materials.

## Results

**Fig. 1:**
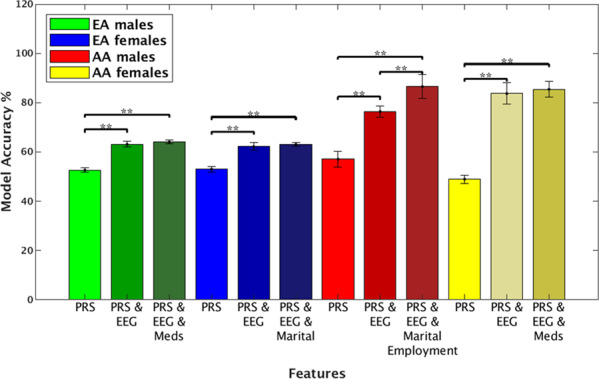
Model accuracy stratified by sex and ancestry. Prediction obtained by only the PRS, the combined EEG and PRS, and features from the highest accuracy scored model for every group (Table 1). Results indicate higher accuracy for the combined feature models suggesting the advantage of adding phenotypes to genetic prediction models. The error bars are standard deviations. *p < 0.05, **p < 0.01.

Significant ML SVM models were calculated predicting remission from AUD for individuals who were previously diagnosed as AUD DSM-5. Sex and ancestry stratified analysis created an individualized model for each of the groups: EA males, EA females, AA males, and AA females (full details of the number of participants and matching age for each of the models in Supplementary Tables S1–S4). Table 1 summarizes the results of the significant predictive model scores across ancestry and sex (see Tables S7–S10 for full results), confirming the previous finding that the combined feature model (e.g., AA males and females models with EEG, PRS, medication, and demographic features) was more accurate than models based on single domain (Fig. 1). We found higher model accuracy when group’s ancestry was defined by genetics than by self-report, in EA males (*p* < 0.001) and AA males (*p* < 0.001) in models with only PRS as features (Supplementary Fig. S1). No difference was found in the females’ groups between the two types of ancestry definition. The AA male group combined feature model of PRS, EEG-FC, marital status, and employment status achieved the highest accuracy of 86.04% (specificity = 85.83%, sensitivity = 86.257%, AUC = 0.97). The AA female group combined feature model of PRS, EEG-FC, and depression medication also achieved high accuracy of 85.43% (specificity = 80.66%, sensitivity = 86.19%, AUC = 0.9). The models of EA male and female groups achieved AUC >0.74 for the model with combined features of PRS, EEG-FC, and medication (accuracy 64.96%, EA males) and PRS, EEG-FC, and marital status (accuracy 63.60%, EA females) (Table 1). Adding discriminatory features to the models increased accuracy, specifically EEG-FC was the most discriminative feature category for all groups (*p* < 0.001).

**Table 1:** Selected models predicting AUD remission stratified by ancestry and sex.

Model [# features]	Specificity (%)	*STD*	Sensitivity (%)	*STD*	Accuracy (%)	*STD*	*AUC*	*STD*
**EA male** PRS, EEG, Other & sleep meds^8^	74.96	0.5	54.82	1.8	64.96	0.9	0.74	0.0
**EA female** PRS, EEG, Marital status^10^	64.38	2.0	62.791	2.3	63.60	1.3	0.77	0.0
**AA male** PRS, EEG, Marital, Employment status^8^	85.83	6.8	86.25	6.4	86.04	5.4	0.97	0.0
**AA female** PRS, EEG, Depression meds^7^	80.66	4.9	90.476	3.1	85.43	3.2	0.98	0.0

Values are means ± standard deviation (*STD*). Alcohol Use Disorder (AUD), European Ancestry (EA), African ancestry (AA). Meds—Medication. Other meds—Any medication that is not one of the seven medications listed in Supplementary materials.

### Discriminative features

Tables 2 and 3 present a summary of selected shared and group-specific features stratified by ancestry and sex for the model with the highest accuracy in each group. Supplementary Tables S11–S14 present the significant features separately for every model.

**Table 2:** Selected discriminative PRS and demographic features predicting AUD remission stratified by ancestry and sex.

	Model	Weight ranking
*PRS, discovery sample*		
Neuroticism, EDU^33^	EA male, EA female	7, 5
Years of education, EDU^33^	EA male, EA female	4, 5
Aggression, EAGLE^35^	EA male	2
Depression, EDU^33^	EA female	3
Max alcohol threshold, MVP^28^	AA male	4
*Demographics*		
Marital status	EA female, AA male	4, 2
Employment status	AA male	2
*Medication*		
Sleep medication	EA male	6
Other medication	EA male	2
Depression medication	AA female	6

*EA* European ancestry, *AA* African ancestry, *PRS* Polygenic Risk Score. Other medication—Any medication that is not one of the seven medications listed in Supplementary materials. Discovery samples are described in Supplementary Table S6.

**Table 3:** Selected discriminative EEG-FC features predicting AUD remission stratified by ancestry and sex.

	Frequency	Model	Weight ranking
*Lower connectivity*			
Isthmus cingulate—Rostral middle frontal	Theta	EA male	3
Parahippocampal—Frontal pole	Alpha	EA female	3
Parahippocampal—Lateral occipital	Theta	EA female	4
Rostral anterior cingulate—Inferior temporal	Theta	AA female	1
Fusiform—Lateral occipital	Theta	EA female	1
Fusiform—Paracentral	Theta	EA female	2
Fusiform—frontal pole	Theta	AA female	3
Precuneus—Posterior cingulate	Gamma	AA female	2
Rostral middle frontal—Inferior temporal	Gamma	EA male	1
Medial orbito frontal—Caudal middle frontal	Gamma	AA male	1
*Higher connectivity*			
Superior Temporal—Lingual	Gamma	EA male	1
Superior Parietal—Insula	Beta	EA female	2
Inferior Parietal—Insula	Theta	AA male	2
Inferior Parietal—Superior temporal	Alpha	EA female	1

*EA* European ancestry, *AA* African ancestry, Lower, Higher coherence of the AUD group compared to the remitted group. Weight ranking—Order of importance in the prediction model according to the beta value.

#### EEG-FC

**Fig. 2:**
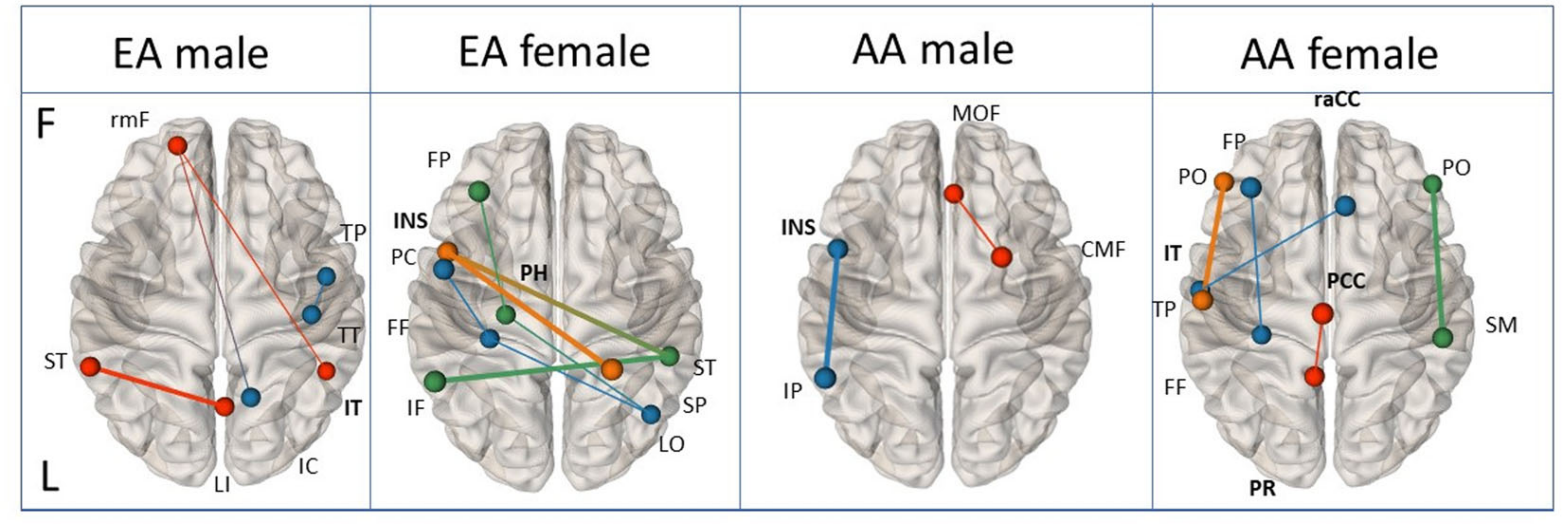
EEG functional connectivity AUD remission biomarkers. AUD remission prediction models reveal ancestry/sex group-specific brain connectivity biomarkers discriminating between those who recovered from AUD to those who did not. Results highlight lower connectivity in theta (blue) and gamma (red) in areas related to DMN (in bold -IT, PCC, PR, raCC, PH) and higher connectivity in theta (blue) and beta (orange) between insula and inferior and superior parietal regions respectively (in bold INS, IP, IT) specific to every sex and ancestry predicting the maintenance of AUD. blue-theta, green-alpha, orange-beta, red-gamma. Thinner lines—lower connectivity, Thicker lines—higher connectivity. CMF Caudal middle frontal, FF fusiform, FP frontal pole, INS insula, IC Isthmus cingulate, IP inferior parietal, IT inferior temporal, LI lingual, LO Lateral Occipital, MOF medial orbito frontal, PC paracentral, PCC posterior cingulate cortex, PH parahippocampus, PO parsorbitalis, PR precuneus, raCC rostral anterior cingulate cortex, SM supramarginal, rmF Rostral middle frontal, TP temporal pole, TT Transverse temporal, SP Superior parietal, ST Superior temporal.

Default Mode Network (DMN) FC, as well as, connectivity levels in other brain networks, were found to discriminate between the groups and to predict AUD remission (selected significance FC features according to groups in Table 3 and Fig. 2. Full list of FC features including right, and left hemispheres can be found in Supplementary Tables S11–S15). Lower connectivity of DMN ROIs was found in the continued AUD group, especially in the range of theta and gamma bands. Known DMN hubs including the precuneus (AA female), the posterior cingulate (AA female), and the middle frontal (EA male, AA male) showed lower gamma connectivity. Lower Theta connectivity was found in other more temporal DMN hubs such as the temporal cortex (AA female) and the parahippocampal formation (EA female) with anterior and posterior brain areas. Lower theta connectivity in the continued AUD versus remitted group was also found between the fusiform and posterior and anterior brain areas. Higher connectivity in the continued AUD group was found in selected temporal and parietal areas including the insula connectivity with superior parietal (beta, EA female) and with inferior parietal (theta, AA male) (Fig. 2).

#### PRS

Significant discriminative shared and specific PRS were found between the groups (selected significance PRS features according to groups in Table 2 and full list can be found in Supplementary Tables S11–S14, including weight ranking). The discriminative features include PRS related to personality traits (neuroticism (EA males, EA females) and aggression (EA male), as well as, depression (EA females), socio-demographic (years of education (EA males, EA females)) and alcohol-related (maximum number of alcoholic drinks within 24 h (AA males)).

#### Medication

Adding medications to the PRS models significantly increased model accuracy for EA males—sleep medication, AA males—other medication. (*p*_(PRS vs PRS & sleep medication)_ <0.001, *p*_(PRS vs PRS & other medication)_ <0.001, respectively). Depression medication increased the accuracy score of the PRS & EEG-FC model but this increase did not reach significance (*p*_(PRS & EEG vs PRS & EEG & depression medication)_ = 0.5). In those cases, taking medication predicted maintenance of the AUD state (Table 2 for weight ranking).

#### Marital status

Marital status feature discriminated between the groups revealing that more members from the remitted AUD EA female and AA male groups were not married compared to their AUD counterparts. (Table 2 for weight ranking). EA female: *p*_(PRS & EEG vs. PRS & EEG & Marital status)_ <0.004, AA male: *p*_(PRS & EEG vs. PRS & EEG & Marital status)_ <0.001.

#### Employment status

Employment status feature discriminated between the groups revealing that more members from the remitted AUD AA male group were not employed compared to their AUD counterparts. (Table 2 for weight ranking). *p*_(PRS & EEG vs. PRS & EEG & Marital & Employment status)_ <0.001.

## Discussion

Of the one-third of individuals with AUD who attempt to quit drinking every year, only 25% are successful in reducing their consumption a year later. Therefore, understanding the parameters that can set an optimal initial state (including biomarkers, demographic, and medications) can greatly affect the success of remission from AUD. Using multimodal, multi-featured machine learning applications with the COGA longitudinal dataset, we uncovered these parameters, uniquely characterized per sex and ancestry. This is the first study to formulate a multimodal-based prediction model to determine AUD individuals who are going to be in remission from AUD. Results confirmed previous results showing that the combined feature model (e.g., EEG, PRS, medication, and demographic information) achieved a higher prediction score than models based on single domain suggesting that genetics prediction models will improve from the addition of phenotypes to the calculation. Intriguingly, results indicate higher accuracy scores for EA and AA males, when the ancestry was defined by genetics than by self-report for models with only PRS features. Several discriminative features were identified for each of the models revealing novel predisposition sex and ancestry-specific AUD remission biomarkers. EEG-FC in all groups was found to distinguish between the continued AUD and the remitted AUD group, revealing DMN and fusiform lower and insula higher functional connectivity in the continued AUD group. Several discriminative PRS were shared (neuroticism PRS and years of education PRS in EA groups), while others PRS were group-specific, such as PRS associated with aggression were important for EA males, and depression PRS were important for EA females. Being married, employed, and taking medication predicted the maintenance of the AUD state. Overall, our findings suggest that wide range of multidimensional features with high internal homogeneity groups will formulate better predictive models.

Our results underscore previous findings showing the high predictive value of neurophysiological brain function to predict/classify neurological disorder^8^. The EEG-FC discriminative features highlight the difference in the neural connectivity underlying resting state spontaneous processes (mind wandering, self-reference, and other introspective processes) between those with continued AUD vs remitted AUD. The continued AUD group showed a lower level of DMN connectivity confirming previous findings of aberrant DMN function in AUD and across psychiatric conditions such as depression, schizophrenia, and autism^44,45^. DMN activity during resting state is implicated in memory consolidation because of the commonality of neural systems to both processes, and, therefore, may be related to aberrant related mental processes such as working memory deficit^46^, inferior cognitive performance, inferior memory formation, and poor learning of cognitive skills^47^ in alcoholics. Indeed the continued AUD EA female group showed lower connectivity of parahippocampal with the anterior and posterior areas of the brain, previously implicated in memory^48^ and cognitive^49^ functions, and the AA males group showed lower connectivity in the precuneus/posterior cingulate, two of the main DMN hubs, which are suggested to play a pivotal role in how the intrinsic activity is mediated throughout the DMN^50^. Both female groups showed lower connectivity involving the fusiform supporting previous fMRI resting-state findings linking cognitive impairment and lower fusiform connectivity^51^. The insula was found to increase connectivity with superior temporal (EA females) and inferior parietal (AA males) in the group that maintains AUD diagnosis. Previous study showed that greater functional coupling between the anterior insula and the left frontoparietal network is linked to smoking and impulsivity^52^. Given the insula’s role in interoceptive awareness and homeostatic processing, this lower activity of connectivity may relate to bias towards immediate rewards^52^ and increased tension^53^ associated with addiction^52^. Interestingly, the structural integrity of the salience network (insula) and DMN lower activity was previously linked to the salience network role as regulating dynamic changes in other networks^54^. This theory suggests that if the salience network in the continued AUD group is damaged it might relate to the DMN lower functioning^55^. Overall, these brain networks’ connectivity showed aberrant functions related to AUD. Observed EEG differences between the genders and between the ancestries support the importance of identifying group-specific prediction models. EEG is a highly heritable phenotype and the differences revealed are the first steps in identifying and distinguishing between different genders and ancestries for the purpose of deepening our knowledge about disease recovery.

The present study reinforces recent discoveries that show the inherent power of adding phenotypes to the genetics prediction model in order to increase accuracy^8^. Several group-specific PRS were identified as distinguishing between the continued AUD and the remitted AUD group. For example, while the EA male model includes PRS related to aggression, the EA female model includes PRS related to depression. Interestingly, both EA group models for the prediction of AUD remission include neuroticism PRS and years of education PRS. Our findings are in line with recently published studies showing PRS association with disorder outcomes in depression^56^, schizophrenia^57^, and alcohol-related phenotypes^58^. These findings highlight the potential of PRS collections to predict the course of development and recovery from diseases. PRS collections representing genetic fingerprint of various phenotypes allow embodying complex diseases with multiple domains. Notably, our results demonstrate the significance of using genetic data over self-report to identify self-ancestry, which increases the genetic homogeneity of the groups, leading to higher prediction scores.

Contrary to our hypothesis, taking medication (EA male: medication for sleep and other, AA female: medication for depression) predicted maintenance of AUD state. Evidence indicates that individuals suffering from comorbidity of other disorders will be disadvantaged in dealing with the physical and psychological processes that accompany withdrawal from addiction^59^. Specifically, alcohol and sleep disturbances have complex mutual relationships as alcohol is used by more than one in ten individuals as a hypnotic agent to self-medicate sleep problems^60^^.^, thus increasing the likelihood of developing alcohol problems^61^. Moreover, studies show that sleep disturbances are extremely common during withdrawal from alcohol dependence and may persist for several months despite continued abstinence^62^, and hence may interfere with remission and contribute to relapse^63^. Contrary to our hypothesis, results showed that marriage and employment status predicted maintenance of the AUD state. Marriage and employment may add additional stressors to the alcoholic’s state of mind that precludes seeking help. Co-workers, spouses, and other family members often experience many tensions and heightened emotional distress caused by the negative consequences of living and working with a person with AUD^64^ leading to a challenging complex environment for the AUD individual. As many studies have noted, therapeutic programs treating married AUD individuals should involve the family/spouse^64^, and taking into account the difficulty for married and employed individuals to leave for rehabilitation for long periods of time, which has led to the development of programs such as Family Systems Therapy (FST)^65^ and Community Reinforcement and Family Training (CRAFT)^66^.

Results indicated higher accuracy for the AA groups over the EA group models. The research of biomarkers, prediction models, and machine learning algorithms rely on group homogeneity and relevant features. Therefore, the higher AA accuracy could be due to better fit of the features to the target and that EA genetic-based ancestry definition has variation leading to the reduction in EA group homogeneity. Studies have shown that EA forms a structured population due to historical immigration of diverse source populations^67^. Future studies might consider dividing EA groups to subgroups according to genetic variation or finding new approaches to define ancestry^68,69^.

Identifying individuals who are ready for the challenge to renounce addiction (and those who are not ready) holds enormous possibilities including intervention and therapy programs. Further, strengthening the AUD individual by altering those biomarkers, psychosocial or demographic “protective” characteristics, can elevate motivation for the initiation of successful remission. Overall, our findings demonstrate the importance of embedded ancestry and sex in the analysis towards the formulation of personalized prediction model. Interestingly, we found that identifying ancestry by genetic data might increase group homogeneity leading to higher accuracy of the prediction model. We further show that the model based on various features from different areas of health (genetics, electrophysiology, medication, and demographic data) outperform prediction models based on features derived from a single domain. We identified specific robust features of PRS and EEG functional connectivity for each sex/ancestry group, further expanding our knowledge of the predisposition biomarkers including genetics and brain mechanisms underlying the process of remission from AUD.

## Limitations

Given the uniqueness of the COGA dataset (with genetics, EEG measurements, and AUD remitter status), analysis on an independent dataset was not available. The latest prediction models’ approach is towards precision medicine, in which sex and ancestral stratification analysis produce more group-specific tailored results. This approach led to different sample sizes, with AA groups showing a smaller cohort. For homogeneous analysis across different group’ sizes, we applied CV analysis on all models, while additional training/testing validation was applied and confirmed the CV results on the larger samples (EA males and females, *p* > 0.1 for all models). Future studies with larger cohorts are required to further validate these results. Another limitation is related to the scope of features. Various symptomatic and psychosocial features were implicated in previous studies as associated with AUD development, including our own work^8^. These features were not included in the current analysis to enable a focus on biomarkers (genetics, brain function) for prediction. Future studies with a wider selection of features are required to further investigate the variables that best predict remission from AUD.

## Supplementary information

SUPPLEMENTAL MATERIAL

## Data Availability

All computer code used to generate the results reported here is freely available and can be accessed by contacting the corresponding author of this article.
