# Supplementary material for: Predicting alcohol use disorder remission: a longitudinal multimodal multi-featured machine learning approach
Source: Transl Psychiatry. 2021 Mar 15;11:166. doi: 10.1038/s41398-021-01281-2 (PMC7960734; doi:10.1038/s41398-021-01281-2)
Supplement: Supplementary file 1 — SUPPLEMENTAL MATERIAL [file 41398_2021_1281_MOESM1_ESM.docx]

**Supplementary Materials for:**

**Predicting Alcohol Use Disorder remission:**

**A longitudinal multimodal multi-featured machine learning approach**

Sivan Kinreich^1^, Vivia V. McCutcheon^2^, Fazil Aliev^3,4^, Jacquelyn L. Meyers^1^, Chella Kamarajan^1^, Ashwini K. Pandey^1^, David B. Chorlian^1^, Jian Zhang^1^, Weipeng Kuang^1^, Gayathri Pandey^1^, Stacey Subbie-Saenz de Viteri^1^, Meredith W. Francis^5^, Grace Chan^6^, Jessica L. Bourdon^2^, Danielle M. Dick^3^, Andrey P. Anokhin^2^, Lance Bauer^6^, Victor Hesselbrock^6^, Marc A. Schuckit^7^, John I. Nurnberger Jr.^8^, Tatiana M. Foroud^9^, Jessica E. Salvatore^10,11^, Kathleen K. Bucholz^2^, Bernice Porjesz^1^

**Supplementary Method**

**Data, participants and experimental protocol:**

COGA GWAS data are available through dbGaP (phs000125.v1.p1; phs000763.v1.p1; phs000976.v1.p1; phs000092.v1.p1; Smokescreen data – pending at dbGaP).

Ascertainment and assessment procedures of COGA recruits have been described elsewhere^1–3^, and are also available at this website: <https://zork5.wustl.edu/niaaa/coga_instruments/resources.html>. COGA data have been collected since 1989, with the support of several NIH grants targeting various objectives. Data from seven collection sites were included in this study (University of Connecticut, Indiana University, University of Iowa, SUNY Downstate, Washington University in St Louis, University of California at San Diego, Howard University. COGA maintains similar protocols for every aspect of the data collection across all of its sites, including recruitment, interviews and experiment protocols^4^. Subjects with a history of hallucinogen abuse (e.g., LSD), hearing or visual impairment, or head injury were excluded from the study. However, given the nature of the disorder, subjects with a history of other substance use and/or Antisocial personality disorder as co-existing conditions and with a history of Conduct disorder, Attention deficit hyperactivity disorder, and Oppositional Defiant Disorder were included. Individuals self-identify was defined by response to the following options: 'Native American/American Indian', 'Asian', 'Pacific Islander', 'African-American/Black', 'Caucasian/White', ‘Hispanic’ and 'Other'.

**Participants**

The AUD and remission phenotypes were based on information from 2 consecutive interviews. Both continued AUD and remitted AUD met criteria for current AUD at the first interview, defined as the presence of 2 or more AUD criteria within the previous 12 months. Remission at the second interview was defined as absence of all AUD criteria other than craving for at least 12 months and either low-risk drinking or abstinence (*n*= 688, 413 males, 275 females, mean age at initial visit: 30.62 ± 9.41, mean number of years between visits = 4.6 ± 1.7). The continued AUD group met criteria for current AUD at both interviews (*n* = 688, 413 males, 275 females, mean age: 30.79 ± 9.36, mean number of years between visits =4.8 ± 1.6). The analysis was done on the data collected during the first visit to predict remission status at the second visit. In a series of analyses, the groups were further divided according to ancestry (EA, AA) and sex (male, female). Stratified analysis by ancestry was done twice: once with ancestry identified by self-report and once identified by implementing SNPrelate^5^ to estimate principal components from GWAS data which subsequently used to determine EA and AA. Sex, ancestry, and features’ missing values dictated a series of analyses that included different subsets of subjects. All groups were matched on age. A full description of each of the groups can be found in Supplementary Tables S1-S4.

**EEG recording system and procedure:**

EEG data collection follows COGA protocols at all sites including equipment and experiment protocols^2,4^. Resting EEG was recorded for four minutes in all participants as they were resting on a comfortable chair in a dimly lit, sound-attenuated RF-shielded booth (Industrial Acoustics, Inc., Bronx, NY, USA). A 64-channel electrode cap (Electro-Cap International, Inc., Eaton, OH, USA) based on the Extended 10–20 System^6,7^ was used. The reference electrode was at the tip of the nose, and the ground electrode was placed at the forehead. The electrooculogram (EOG) was recorded by a supraorbital vertical electrode and by a horizontal electrode on the external canthus of the left eye. Electrode impedances were maintained below 5 kΩ. Electrical activity was amplified 10,000 times using SynAmps2 amplifiers (Compumedics, Charlotte, NC) and was recorded continuously over a bandwidth between near-DC (0 Hz) and 100.0 Hz on a Neuroscan system (Versions 4.3–4.5; Compumedics USA, Charlotte, NC) at a sampling rate of 500 or 512 Hz. Participants were asked to stay awake with eyes closed and not to move.

**EEG preprocessing:**

Matlab (Mathworks Inc, Natick, MA), EEGLAB^8^ and Python were used for all calculations. The continuous EEG data was low-pass filtered with a cutoff of 60 Hz to reduce motor artifacts, and a digital notch filter was applied at 60 Hz to remove artifacts caused by alternating current line noise.

**Functional connectivity MNE calculation**

**Source construction**

The MNE-Python^9,10^ package is an open-source tool for electrophysiology analysis, visualization, and data representation in Python. The general procedures for the MNE source localization have been described in detail elsewhere^10,11^ and in supplemental materials. Briefly, 68 cortical regions were defined with the FreeSurfer “aparc” parcellation. FreeSurfer parcellation scheme (aparc.lh/rh), based on the Desikan–Killiany Atlas^12^, was used to extract 68 cortical regions from both hemispheres. A list of regions is given in Table 5. The continuous data is cut up into segments in a short time window and are referred to as “epochs”. For inverse source reconstruction and beamforming, we first computed the forward solution. We computed source space of dipole locations, a conductor model for the head, and the sensor locations relative to those dipoles (using a template provided by MNE, with three layers of inner skull, outer skull, and outer skin). In practice, this means that the BEM surfaces and source space must be coregistered with the EEG sensors, which are digitized in the Neuromag head coordinate frame (defined by the digitizednasion, LPA, and RPA). dSPM^13^ was used for source localization with loose orientation of 0.2, depth weighting of 0.8^14^, and SNR value of 1.0. No specific regularization was used in the beamformer filter estimation. The inverse operator is determined and applied to each of the epochs. We computed the spectral coherence to measure functional connectivity (FC) between EEG signals of 68 ROIs at a specific frequency band f. The connectivity matrix of size 68 × 68 where the entries contain the value of the spectral coherence between the EEG signals of ROIs at the frequency f. The connectivity matrices are computed at each and across theta (4 – 8Hz), alpha (8 – 12Hz), beta (12 – 30Hz), and gamma (30 – 60Hz) frequency bands.

**Genome-Wide Polygenic Risk Scores. PRS calculations**

Genome-wide association estimates from samples and phenotypes was used to construct genome-wide polygenic scores in the target samples. Target samples are filtered with MAF<=0.005 and HWE<=10^-6. The algorithm first removes palindromic SNPs (which can be ambiguous with respect to the reference allele when going across samples), Clump was used and score procedures in PLINK to sum each individual’s total number of minor alleles from the score SNPs, with each SNP weighted by the negative log of the GWAS association p value and sign of the association (beta) statistic. Clumping was done with respect to the linkage disequilibrium (LD) pattern in the 1000 Genome Phase 3 sample using a 500kb physical distance and an LD threshold of r2>=0.25. Each sample (European ancestry (EA), African ancestry (AA) and All) uses corresponding genotypes from 1000G, for example, for AA individuals 1000G AA individuals were used. Thus, the polygenic scores were constructed of SNPs that capture independent genetic association signals from the discovery GWAS. A series of scores in COGA was calculated, that included SNPs meeting increasingly stringent p-value thresholds from the discovery GWAS sample (*P*<.0001, *P*<0.001, *P*<0.01, *P*<0.05, *P*<0.10, *P*<0.20, *P*<0.30, *P*<0.40, *P*<0.50).

**Machine learning preprocessing**

We used the least absolute shrinkage and selection operator (LASSO) penalty approach shown by Tibshirani^15^ for feature selection. The sparsity property of LASSO which generates coefficient estimates of exactly zero, shrinks the estimation variance resulting with a more interpretable model^16^. Previous use of this application for genomic data^17^ has shown that selective number of discriminating features can reach satisfactory classification. Regularization parameters were determined using a tenfold cross-validation (CV) procedure, with the label: continued AUD vs. remitted AUD as the response variable. The reduced set of the most discriminant features with non-zero coefficient was fed into the model to predict participants status to either continued AUD group or remitted AUD group. A supervised linear-kernel SVM that included parameter optimization was trained with a tenfold CV procedure to classify participants into the two groups. The tenfold CV procedure involved randomly dividing the participants into ten equal groups, training the classifier on nine of them and tested the trained model on the left out one. To ensure randomization of the participants in the calculated model, the dataset was shuffled before every fold. To take advantage of the randomization procedure, we repeated this process ten times, averaging the output results. CV was applied to all models with additional training/testing (70:30) validation analysis to confirm results in the larger samples (EA male and females). Model performance was evaluated by calculating the number of true positives (TP, number of correctly classified remitted AUD) and true negatives (TN, number of correctly classified continued AUD) scores. We computed the classification accuracy as the ratio of sum of TP and TN divided by the sum of all classified subjects. Area under curve (AUC)^18^ was used to evaluate the classification models. To control for **multicollinearity**all model feature’s correlations did not exceed R=0.5 (average 0.24+-0.19).

**COGA’s features assessment**

**Marital status –**

MARRIED - 1

LIVING AS MARRIED -1

WIDOWED -0

SEPARATED -0

DIVORCED – 0

NEVER MARRIED - 0

**Employment status** – 1/0

**Medication intake -** In the last 30 days, have you taken prescription medications for two weeks or longer… IF YES, ASK: What did you take? 1/0

1. To make you feel less nervous?

2. To help you sleep

3. To feel less depressed?

4. For headaches?

5. To have more energy

6. Women Only: For birth control?

7. Containing steroids?

8. For anything else? (Other medication)


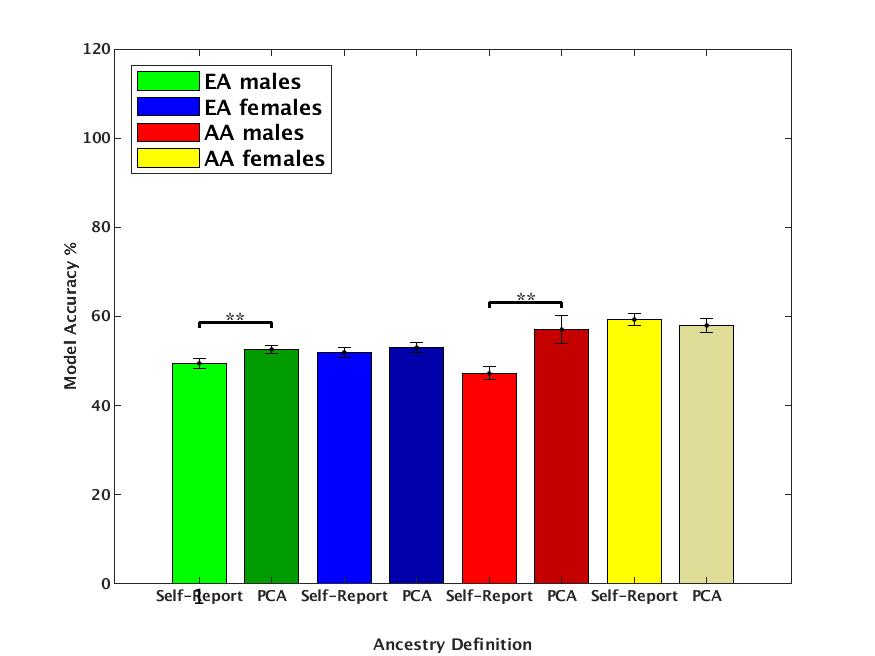


Self-Report Genetics Self-Report Genetics Self-Report Genetics Self-Report Genetics

**Figure S1.** Model accuracy stratified by sex and ancestry. Results indicate higher model accuracy when the ancestry was defined by genetics (ancestral principal components [PCA]) rather than by self-report for the males’ groups.

**p* < .05, ***p* < .01.

| **Table S1: EA male group, number of participants and difference in *p* values between the two groups mean age for each of models** | | | | | | | |
| --- | --- | --- | --- | --- | --- | --- | --- |
|  | Continued AUD | | | Remitted AUD | | | |
| Model Features | # Subjects | *Mean age* | *STD* | # Subjects | *Mean age* | *STD* | *P values of age* |
| PRS | 348 | 32.92 | 11.44 | 348 | 32.58 | 11.23 | 0.69 |
| PRS, Anxiety meds | 348 | 32.92 | 11.44 | 348 | 32.58 | 11.23 | 0.69 |
| PRS, Sleep meds | 348 | 32.92 | 11.44 | 348 | 32.58 | 11.23 | 0.69 |
| PRS, Depression meds | 348 | 32.92 | 11.44 | 348 | 32.58 | 11.23 | 0.69 |
| PRS, Headache meds | 348 | 32.92 | 11.44 | 348 | 32.58 | 11.23 | 0.69 |
| PRS, Energy meds | 348 | 32.92 | 11.44 | 348 | 32.58 | 11.23 | 0.69 |
| PRS, with Steroids meds | 348 | 32.92 | 11.44 | 348 | 32.58 | 11.23 | 0.69 |
| PRS, Other meds | 348 | 32.92 | 11.44 | 348 | 32.58 | 11.23 | 0.69 |
| PRS, Employment status | 300 | 32.90 | 11.57 | 300 | 32.31 | 11.27 | 0.52 |
| PRS, Marital status | 337 | 32.89 | 11.47 | 343 | 32.64 | 11.24 | 0.76 |
| EEG | 151 | 32.56 | 11.46 | 127 | 31.55 | 10.69 | 0.45 |
| PRS, EEG | 151 | 32.56 | 11.46 | 127 | 31.55 | 10.69 | 0.45 |
| PRS, EEG, Sleep meds | 151 | 33.58 | 12.17 | 127 | 32.98 | 11.88 | 0.68 |
| PRS, EEG, Other meds | 151 | 32.56 | 10.96 | 127 | 32.07 | 11.38 | 0.71 |
| PRS, EEG, Other, Sleep meds | 151 | 32.33 | 10.63 | 127 | 31.86 | 10.52 | 0.71 |
| PRS, EEG, Employment status | 134 | 32.59 | 11.31 | 113 | 33.90 | 10.70 | 0.35 |
| PRS, EEG, Marital status | 141 | 32.70 | 11.30 | 122 | 32.13 | 11.18 | 0.68 |
| PRS, EEG, Anxiety meds | 151 | 33.41 | 11.37 | 127 | 31.76 | 10.49 | 0.21 |
| PRS, EEG, Headache meds | 151 | 32.62 | 11.39 | 127 | 32.41 | 11.04 | 0.87 |
| PRS, EEG, Sleep meds | 151 | 33.25 | 11.36 | 127 | 33.14 | 11.98 | 0.93 |
| *Note:* Values are means ± standard deviation (*STD*). Alcohol Use Disorder (AUD). | | | | | | | |

| **Table S2: EA female group, number of participants and difference in *p* values between the two groups mean age for each of models** | | | | | | | |
| --- | --- | --- | --- | --- | --- | --- | --- |
|  | Continued AUD | | | Remitted AUD | | |  |
| Model | # Subjects | *Mean age* | *STD* | # Subjects | *Mean age* | *STD* | *P values of age* |
| PRS | 238 | 28.8 | 8.88 | 238 | 28.49 | 8.70 | 0.69 |
| PRS, Anxiety meds | 238 | 28.8 | 8.88 | 238 | 28.49 | 8.70 | 0.69 |
| PRS, Sleep meds | 238 | 28.8 | 8.88 | 238 | 28.49 | 8.70 | 0.69 |
| PRS, Depression meds | 238 | 28.8 | 8.88 | 238 | 28.49 | 8.70 | 0.69 |
| PRS, Headache meds | 238 | 28.8 | 8.88 | 238 | 28.49 | 8.70 | 0.69 |
| PRS, Energy meds | 238 | 28.8 | 8.88 | 238 | 28.49 | 8.70 | 0.69 |
| PRS, Birth control | 238 | 28.8 | 8.88 | 238 | 28.49 | 8.70 | 0.69 |
| PRS, With Steroids meds | 238 | 28.8 | 8.88 | 238 | 28.49 | 8.70 | 0.69 |
| PRS, Other meds | 238 | 28.8 | 8.88 | 238 | 28.49 | 8.70 | 0.69 |
| PRS, Employment status | 209 | 28.80 | 9.01 | 208 | 28.37 | 8.46 | 0.61 |
| PRS, Marital status | 226 | 28.95 | 8.80 | 230 | 28.65 | 8.64 | 0.71 |
| EEG | 84 | 31 | 10.26 | 95 | 28.11 | 9.40 | 0.05 |
| PRS, EEG | 84 | 31 | 10.26 | 95 | 28.11 | 9.40 | 0.05 |
| PRS, EEG, Headache meds | 84 | 28.08 | 8.94 | 95 | 28.35 | 8.80 | 0.83 |
| PRS, EEG, Employed | 77 | 28.29 | 8.49 | 92 | 28.79 | 8.52 | 0.70 |
| PRS, EEG, Married | 77 | 29.11 | 9.09 | 94 | 27.74 | 8.21 | 0.30 |
| PRS, EEG, Depression meds | 84 | 27.41 | 8.07 | 95 | 29.81 | 9.69 | 0.07 |
| PRS, EEG, Energy meds | 84 | 29.15 | 8.66 | 95 | 28.11 | 8.31 | 0.41 |
| PRS, EEG, Energy, Anxiety meds | 84 | 29.80 | 8.89 | 95 | 27.67 | 8.79 | 0.10 |
| PRS, EEG, Energy, Depression meds | 77 | 30.10 | 8.67 | 92 | 29.01 | 8.50 | 0.41 |
| *Note:* Values are means ± standard deviation (*STD*). Alcohol Use Disorder (AUD). | | | | | | | |

| **Table S3: AA male group, number of participants and difference in *p* values between the two groups mean age for each of models** | | | | | | | |
| --- | --- | --- | --- | --- | --- | --- | --- |
|  | Continued AUD | | | Remitted AUD | | |  |
| Model Features | # Subjects | *Mean age* | *STD* | # Subjects | *Mean age* | *STD* | *P values of age* |
| PRS | 65 | 31.47 | 9.42 | 65 | 31.09 | 9.56 | 0.81 |
| PRS, Anxiety meds | 65 | 31.47 | 9.42 | 65 | 31.09 | 9.56 | 0.81 |
| PRS, Sleep meds | 65 | 31.47 | 9.42 | 65 | 31.09 | 9.56 | 0.81 |
| PRS, Depression meds | 65 | 31.47 | 9.42 | 65 | 31.09 | 9.56 | 0.81 |
| PRS, Headache meds | 65 | 31.47 | 9.42 | 65 | 31.09 | 9.56 | 0.81 |
| PRS, With Steroids meds | 65 | 31.47 | 9.42 | 65 | 31.09 | 9.56 | 0.81 |
| PRS, Other meds | 65 | 31.47 | 9.42 | 65 | 31.09 | 9.56 | 0.81 |
| PRS, PRS, Employment status | 46 | 31.43 | 9.97 | 47 | 30.97 | 9.23 | 0.81 |
| PRS, Marital status | 63 | 31.68 | 9.50 | 65 | 31.09 | 9.56 | 0.72 |
| PRS, EEG | 20 | 30.95 | 8.49 | 23 | 32.52 | 10.93 | 0.60 |
| PRS, EEG, Depression meds | 20 | 28.35 | 8.52 | 23 | 31.56 | 10.21 | 0.27 |
| PRS, EEG, Anxiety meds | 20 | 31.65 | 9.77 | 23 | 31.60 | 10.29 | 0.98 |
| PRS, EEG, Sleep meds | 20 | 30.35 | 9.93 | 23 | 32.17 | 9.37 | 0.53 |
| PRS, EEG, Depression meds | 20 | 31.25 | 9.40 | 23 | 31.69 | 10.08 | 0.88 |
| PRS, EEG, Employment status | 14 | 29.85 | 8.42 | 16 | 34.50 | 9.57 | 0.17 |
| PRS, EEG, Marital status | 19 | 31.63 | 9.25 | 23 | 30.73 | 10.04 | 0.76 |
| PRS, EEG, Anxiety meds | 19 | 29.89 | 8.10 | 23 | 31.08 | 9.34 | 0.66 |
| PRS, EEG, Anxiety, Sleep meds | 20 | 32.60 | 11.25 | 23 | 34.39 | 9.13 | 0.56 |
| PRS, EEG, Marital status, Anxiety meds | 19 | 33.47 | 7.98 | 23 | 32.30 | 10.06 | 0.68 |
| *Note:* Values are means ± standard deviation (*STD*). Alcohol Use Disorder (AUD). | | | | | | | |

| **Table S4. AA female group, number of participants and difference in *p* values between the two groups mean age for each of models** | | | | | | | |
| --- | --- | --- | --- | --- | --- | --- | --- |
|  | Continued AUD | | | Remitted AUD | | |  |
| Model Features | # Subjects | *Mean age* | *STD* | # Subjects | *Mean age* | *STD* | *P values of age* |
| PRS | 37 | 30.32 | 8.13 | 37 | 29.97 | 7.71 | 0.84 |
| PRS, Anxiety meds | 37 | 30.32 | 8.13 | 37 | 29.97 | 7.71 | 0.84 |
| PRS, Sleep meds | 37 | 30.32 | 8.13 | 37 | 29.97 | 7.71 | 0.84 |
| PRS, Depression meds | 37 | 30.32 | 8.13 | 37 | 29.97 | 0.84 | 0.84 |
| PRS, Headache meds | 37 | 30.32 | 8.13 | 37 | 29.97 | 7.71 | 0.84 |
| PRS, Energy meds | 37 | 30.32 | 8.13 | 37 | 29.97 | 7.71 | 0.84 |
| PRS, Birth control | 37 | 30.32 | 8.13 | 37 | 29.97 | 7.71 | 0.84 |
| PRS, With steroids meds | 37 | 30.32 | 8.13 | 37 | 29.97 | 7.71 | 0.84 |
| PRS, Other meds | 37 | 30.32 | 8.13 | 37 | 29.97 | 7.71 | 0.84 |
| PRS, Employment status | 25 | 30.52 | 8.17 | 22 | 30.27 | 7.82 | 0.91 |
| PRS, Marital status | 37 | 30.32 | 8.13 | 36 | 30.08 | 7.79 | 0.89 |
| EEG | 16 | 29.18 | 7.30 | 22 | 28.86 | 6.86 | 0.88 |
| PRS, EEG | 16 | 29.18 | 7.30 | 22 | 28.86 | 6.86 | 0.88 |
| PRS, EEG, Sleep meds | 16 | 32.37 | 9.32 | 22 | 28.63 | 7.31 | 0.17 |
| PRS, EEG, Energy meds | 16 | 29.25 | 7.72 | 22 | 30.09 | 7.30 | 0.73 |
| PRS, EEG, Anxiety meds | 16 | 33.87 | 7.21 | 22 | 29.72 | 8.08 | 0.11 |
| PRS, EEG, Sleep meds | 16 | 29.75 | 7.39 | 22 | 31.72 | 7.81 | 0.43 |
| PRS, EEG, Anxiety meds | 16 | 30.75 | 8.45 | 22 | 29.77 | 7.89 | 0.71 |
| PRS, EEG, Headache meds | 16 | 29.68 | 8.40 | 22 | 29.40 | 7.40 | 0.91 |
| PRS, EEG, Energy meds | 16 | 30.06 | 9.12 | 22 | 30.95 | 7.77 | 0.74 |
| *Note:* Values are means ± standard deviation (*STD*). Alcohol Use Disorder (AUD). | | | | | | | |

| **Table S5: Cortical surface of the frontal (F), temporal (T), parietal (P) or occipital (O) lobe areas according to the Desikan–Killiany (DK) Atlas.** | |
| --- | --- |
| Region label | Lobe |
| Caudal anterior cingulate | F |
| Caudal middle frontal | F |
| Frontal pole | F |
| Insula | F |
| Isthmus cingulate | F |
| Lateral orbitofrontal | F |
| Medial orbitofrontal | F |
| Parsopercularis | F |
| Parsorbitalis | F |
| Parstriangularis | F |
| Precentral | F |
| Rostral anterior cingulate | F |
| Rostral middle frontal | F |
| Superior frontal | F |
| Banksts | T |
| Entorhinal | T |
| Fusiform | T |
| Inferior temporal | T |
| Middle temporal | T |
| Parahippocampal | T |
| Superior temporal | T |
| Temporal pole | T |
| Transverse temporal | T |
| Inferior parietal | P |
| Paracentral | P |
| Postcentral | P |
| Posterior cingulate | P |
| Precuneus | P |
| Superior parietal | P |
| Supramarginal | P |
| Cuneus | O |
| Lateral occipital | O |
| Lingual | O |
| Pericalcarine | O |

| **Table S6. PRS Discovery Samples** | | | |
| --- | --- | --- | --- |
| **Sample Name** | **# PRS** | **Phenotypes** | **Sample Size N** |
| MVP^19^ | 6 | "EA_AUD","EA_AUDIT_C","EA_MAX_ALC","AA_AUD",  "AA_AUDIT_C",  "AA_MAX_ALC" | 274,424 |
| EDU^20^ | 6 | "DS_Full","EduYears_Main","EduYears_Main_Men",  "EduYears_Main_Women",  "Neuroticism_Full","SWB_Full" | 293,723 |
| SSGAC^21^ | 3 | "EPRSducational_Attainment","Subjective_WB",  "Cognitive_Performance" | 257,828 |
| ANGST (Anxiety Neuro Genetics Study)^22^ | 1 | "Anxiety" | 17,310 |
| Broad_ABC ([Web: http://broadabc.ctglab.nl/projects](http://broadabc.ctglab.nl/projects)) | 3 | "METAL_combined","METAL_females",  "METAL_males" | N>100.000 |
| CHARGE^23^ | 6 | "men_continuous_alcohol","men_dichotomous_alcohol",  "women_continuous_alcohol","women_dichotomous_alcohol",  "pooled_continuous_alcohol","pooled_dichotomous_alcohol" | 42,900  47,720 |
| EAGLE (Aggression)^24^ | 1 | "EAGLE_Agression" | 18,988 |
| ENIGMA^25^ | 7 | "ICV_ENIGMA","alphaCz","alphaOcc","betaCz","deltaCz",  "peakOcc","thetaCz" | 13,171 |
| Environmental Sensitivity^26^ | 1 | "Env_Sens" | 1,026 MZ twin pairs |
| GELERNTER^27^ | 2 | "EA_ALCDEP","AA_ALCDEP" | 16,087 |
| GSCAN^28^ | 5 | "All_CigarettesPerDay","All_SmokingInitiation",  "All_SmokingCessation","All_DrinksPerWeek","All_AgeOfInitiation" | 941,280 |
| UK_BIOBANK^29^ | 6 | "Alcohol","Fluid_intelligence","Tobacco_use","2018AUDIT_T",  "2018AUDIT_C","2018AUDIT_P" | 108,818 |

| **Table S7. Predicting remission from AUD based on different features in the EA male sample. Ancestry is based on genetic data** | | | | | | | | |
| --- | --- | --- | --- | --- | --- | --- | --- | --- |
| Model [# features] | Specificity  (%) | *STD* | Sensitivity  (%) | *STD* | Accuracy  (%) | *STD* | *AUC* | *STD* |
| PRS [3] | 49.79 | 0.99 | 55.31 | 1.58 | 52.53 | 0.95 | 0.56 | 0 |
| PRS, Anxiety meds [4] | 47.21 | 0.81 | 59.89 | 0.79 | 53.51 | 0.64 | 0.57 | 0 |
| PRS, Sleep meds [4] | 48.06 | 0.89 | 61.18 | 1.1 | 54.55 | 0.54 | 0.57 | 0 |
| PRS, Depression meds [4] | 47.65 | 0.99 | 56.41 | 1.5 | 52.00 | 0.77 | 0.5 | 0 |
| PRS, Headache meds [4] | 46.19 | 1.3 | 60.79 | 1.4 | 53.44 | 0.94 | 0.57 | 0 |
| PRS, Energy meds [4] | 49.69 | 1.46 | 55.37 | 1.79 | 52.51 | 1.21 | 0.56 | 0 |
| PRS, with Steroids meds [4] | 49.35 | 0.87 | 57.55 | 1.42 | 53.42 | 0.7 | 0.56 | 0 |
| PRS, Other meds [4] | 39.79 | 1.22 | 67.58 | 1.20 | 53.5 | 0.65 | 0.58 | 0.00 |
| PRS, Employment status [4] | 50.66 | 1.65 | 45.57 | 2.5 | 48.13 | 0.79 | 0.50 | 0.04 |
| PRS, Marital status [4] | 53.34 | 2.10 | 52.23 | 0.89 | 52.79 | 1.04 | 0.5 | 0 |
| EEG [3] | 82 | 1.5 | 44 | 1.6 | 62.0 | 0.8 | 0.71 | 0 |
| PRS, EEG [6] | 74.32 | 2.13 | 52.0 | 2.11 | 63.24 | 1.81 | 0.72 | 0 |
| PRS, EEG, Marital status, Sleep meds [8] | 73.19 | 1.56 | 53.07 | 1.50 | 63.2 | 1.27 | 0.72 | 0 |
| PRS, EEG, Other meds [7] | 75.60 | 1.53 | 53.9 | 1.8 | 64.8 | 0.98 | 0.74 | 0 |
| PRS, EEG, Other, Sleep meds [8] | 74.96 | 0.58 | 54.82 | 1.86 | 64.9 | 0.90 | 0.74 | 0 |
| PRS, EEG, Employment status [7] | 75.95 | 2.30 | 46.53 | 2.65 | 61.34 | 2.1 | 0.72 | 0 |
| PRS, EEG, Marital status [7] | 71.59 | 3.33 | 48 | 2.80 | 59.87 | 1.85 | 0.71 | 0 |
| PRS, EEG, Anxiety meds [7] | 71.13 | 0.82 | 52.1 | 2.15 | 61.72 | 0.90 | 0.72 | 0 |
| PRS, EEG, Headache meds [7] | 72.55 | 1.8 | 54.0 | 2.31 | 63.3 | 1.17 | 0.7 | 0 |
| PRS, EEG, Sleep meds [7] | 74.11 | 1.74 | 53.42 | 2.0 | 63.83 | 1.29 | 0.7 | 0 |
| *Note:* Standard deviation (*STD*), Area under the curve (*AUC*). | | | | | | | | |

| **Table S8. Predicting remission from AUD based on different features in the EA female sample. Ancestry is based on genetic data** | | | | | | | | |
| --- | --- | --- | --- | --- | --- | --- | --- | --- |
| Model [# features] | Specificity  (%) | *STD* | Sensitivity  (%) | *STD* | Accuracy  (%) | *STD* | *AUC* | *STD* |
| PRS [3] | 61.49 | 1.85 | 44.2 | 2.01 | 52.99 | 1.65 | 0.57 | 0 |
| PRS, Anxiety meds [4] | 71.40 | 3.09 | 29.7 | 2.32 | 50.90 | 1.03 | 0.56 | 0.04 |
| PRS, Sleep meds [4] | 62.51 | 1.81 | 41.45 | 0.95 | 52.16 | 1.25 | 0.57 | 0 |
| PRS, Depression meds [4] | 66.42 | 1.69 | 38 | 2.43 | 52.45 | 1.11 | 0.58 | 0 |
| PRS, Headache meds [4] | 54.01 | 2.98 | 51.5 | 2.98 | 52.77 | 2.04 | 0.57 | 0 |
| PRS, Energy meds [4] | 56.61 | 1.79 | 48.8 | 2.37 | 52.77 | 1.02 | 0.57 | 0 |
| PRS, Birth control [4] | 56.61 | 2.69 | 44.95 | 3.07 | 50.88 | 1.61 | 0.55 | 0.04 |
| PRS, With Steroids meds [4] | 55.65 | 2.14 | 47.25 | 1.91 | 51.52 | 1.71 | 0.57 | 0 |
| PRS, Other meds [4] | 54.97 | 2.13 | 43.25 | 2.22 | 49.21 | 1.36 | 0.53 | 0.05 |
| PRS, Employment status [4] | 57.05 | 1.61 | 45.11 | 1.87 | 51.18 | 1.30 | 0.58 | 0 |
| PRS, Marital status [4] | 56.04 | 3.25 | 44.6 | 3.01 | 50.46 | 2.21 | 0.56 | 0 |
| EEG (6) | 57.59 | 1.7 | 67 | 1 | 60.46 | 1 | 0.75 | 0 |
| PRS, EEG [9] | 63.03 | 2.37 | 61.37 | 1.55 | 62.22 | 1.68 | 0.76 | 0 |
| PRS, EEG, Headache meds [10] | 62.78 | 2.16 | 61.83 | 1.93 | 62.32 | 1.49 | 0.76 | 0 |
| PRS, EEG, Employed [10] | 60.41 | 2.18 | 59.04 | 3.08 | 59.74 | 1.25 | 0.77 | 0 |
| PRS, EEG, Married [10] | 64.38 | 2.04 | 62.79 | 2.38 | 63.60 | 1.32 | 0.77 | 0 |
| PRS, EEG, Depression meds [10] | 62.40 | 2.23 | 60.57 | 1.21 | 61.50 | 1.18 | 0.76 | 0 |
| PRS, EEG, Energy meds [10] | 60.75 | 1.57 | 63.7 | 2.18 | 62.25 | 1.42 | 0.76 | 0 |
| PRS, EEG, Energy, Anxiety meds [11] | 61.13 | 1.34 | 62.06 | 1.53 | 61.59 | 1.19 | 0.76 | 0 |
| PRS, EEG, Energy, Depression meds [11] | 63.97 | 2.33 | 62.26 | 2.44 | 63.13 | 1.86 | 0.78 | 0 |
| *Note:* Standard deviation (*STD*), Area under the curve (*AUC*). | | | | | | | | |

| **Table S9. Predicting remission from AUD based on different features in the AA male sample. Ancestry is based on genetic data** | | | | | | | | |
| --- | --- | --- | --- | --- | --- | --- | --- | --- |
| Model [# features] | Specificity  (%) | *STD* | Sensitivity  (%) | *STD* | Accuracy  (%) | *STD* | *AUC* | *STD* |
| PRS [4] | 58.39 | 3.57 | 54.73 | 3.18 | 56.54 | 2.68 | 0.67 | 0 |
| PRS, Anxiety meds [5] | 56.96 | 1.56 | 58.97 | 2.21 | 57.97 | 0.96 | 0.69 | 0 |
| PRS, Sleep meds [5] | 55.17 | 4.24 | 59.12 | 2.19 | 57.18 | 2.57 | 0.70 | 0 |
| PRS, Depression meds [5] | 57.5 | 2.19 | 57.54 | 2.71 | 57.52 | 2.17 | 0.71 | 0 |
| PRS, Headache meds [4] | 58.03 | 3.06 | 55.43 | 3.32 | 56.72 | 1.97 | 0.67 | 0 |
| PRS, With Steroids meds [5] | 59.64 | 3.87 | 54.56 | 2.67 | 57.06 | 2.43 | 0.69 | 0 |
| PRS, Other meds [5] | 62.85 | 1.84 | 55.08 | 2.64 | 58.91 | 1.52 | 0.69 | 0 |
| PRS, Employment status [5] | 61.22 | 2.68 | 57 | 3.07 | 59.07 | 1.59 | 0.71 | 0 |
| PRS, Marital status [5] | 52.40 | 2.62 | 70.35 | 2.67 | 61.51 | 2.08 | 0.72 | 0 |
| EEG [2] | 60 | 4.3 | 90 | 2.5 | 75.9 | 2.2 | 0.82 | 0 |
| PRS, EEG [6] | 66.31 | 2.71 | 85.21 | 2.24 | 76.08 | 1.51 | 0.91 | 0 |
| PRS, EEG, Depression meds [7] | 66.8 | 3.55 | 86.08 | 2.75 | 76.61 | 2.06 | 0.91 | 0 |
| PRS, EEG, Anxiety meds [7] | 65.78 | 2.77 | 85.21 | 3.66 | 75.65 | 2.30 | 0.91 | 0 |
| PRS, EEG, Sleep meds [7] | 67.36 | 2.21 | 84.34 | 3.04 | 75.99 | 1.82 | 0.91 | 0 |
| PRS, EEG, Marital status, Depression meds [8] | 73.88 | 5.88 | 89.13 | 4.22 | 81.62 | 3.69 | 0.96 | 0 |
| PRS, EEG, Employment status [7] | 63.07 | 4.86 | 78.12 | 3.294 | 70.84 | 3.33 | 0.93 | 0 |
| PRS, EEG, Marital status [7] | 73.88 | 5.88 | 91.30 | 4.58 | 82.73 | 4.52 | 0.96 | 0 |
| PRS, EEG, Marital, employment status [8] | 84.16 | 4.73 | 84.37 | 6.751 | 84.27 | 3.96 | 0.97 | 0 |
| PRS, EEG, Employment, Anxiety meds [8] | 65.38 | 7.47 | 76.87 | 3.019 | 71.22 | 3.81 | 0.93 | 0.00 |
| PRS, EEG, Marital status, Sleep meds [8] | 75 | 3.92 | 90.43 | 3.43 | 82.83 | 2.60 | 0.96 | 0 |
| *Note:* Standard deviation (*STD*), Area under the curve (*AUC*). | | | | | | | | |

| **Table S10. Predicting remission from AUD based on different features in the AA female sample. Ancestry is based on genetic data** | | | | | | | | |
| --- | --- | --- | --- | --- | --- | --- | --- | --- |
| Model [# features] | Specificity  (%) | *STD* | Sensitivity  (%) | *STD* | Accuracy  (%) | *STD* | *AUC* | *STD* |
| PRS [2] | 58.75 | 3.84 | 58.52 | 4.03 | 58.63 | 3.35 | 0.65 | 0 |
| PRS, Anxiety meds [3] | 57.5 | 5.92 | 54.7 | 4.20 | 56.14 | 3.1 | 0.67 | 0 |
| PRS, Sleep meds [3] | 60.98 | 3.97 | 52.05 | 4.60 | 56.62 | 3.87 | 0.6 | 0 |
| PRS, Depression meds [3] | 59.06 | 2.73 | 57.35 | 3.46 | 58.23 | 2.76 | 0.66 | 0 |
| PRS, Headache meds [3] | 59.68 | 4.02 | 56.76 | 3.41 | 58.26 | 2.98 | 0.66 | 0 |
| PRS, Energy meds [3] | 57.81 | 3.68 | 56.17 | 5.79 | 57.01 | 3.71 | 0.6 | 0.1 |
| PRS, Birth control [3] | 58.43 | 6.25 | 58.82 | 4.80 | 58.62 | 4.10 | 0.7 | 0 |
| PRS, With steroids meds [3 | 57.81 | 3.96 | 57.64 | 3.97 | 57.73 | 2.80 | 0.66 | 0 |
| PRS, Other meds [3] | 47.18 | 4.28 | 54.76 | 3.16 | 50.8 | 1.95 | 0.67 | 0 |
| PRS, Employment status [3] | 43.6 | 3.83 | 43 | 5.37 | 43.32 | 3.53 | 0.39 | 0.08 |
| PRS, Marital status [3] | 63.75 | 4.46 | 54.84 | 3.62 | 59.4 | 1.70 | 0.69 | 0 |
| EEG [5] | 72.35 | 6.8 | 89 | 2.3 | 81.79 | 4.09 | 0.97 | 0 |
| PRS, EEG [6] | 78.66 | 6.12 | 88.57 | 4.01 | 83.69 | 4.34 | 0.98 | 0 |
| PRS, EEG, Sleep meds [7] | 81.33 | 9.83 | 89.04 | 2.3 | 85.08 | 5.27 | 0.98 | 0 |
| PRS, EEG, Energy meds [7] | 78.66 | 7.56 | 89.52 | 2.00 | 83.94 | 3.91 | 0.9 | 0 |
| PRS, EEG, Anxiety meds [7] | 79.33 | 3.78 | 88.09 | 3.36 | 83.59 | 1.95 | 0.98 | 0 |
| PRS, EEG, Marital status [7] | 84.66 | 7.06 | 83.5 | 2.41 | 84.1 | 3.83 | 0.98 | 0 |
| PRS, EEG, Depression meds [7] | 80.66 | 4.91 | 90.476 | 3.17 | 85.43 | 3.24 | 0.98 | 0 |
| PRS, EEG, Headache meds [7] | 73.33 | 8.31 | 84.762 | 3.01 | 78.88 | 4.89 | 0.98 | 0 |
| PRS, EEG, Birth control meds  [7] | 70.66 | 3.44 | 86.19 | 4.17 | 78.21 | 2.48 | 0.9 | 0 |
| *Note:* Standard deviation (*STD*), Area under the curve (*AUC*), meds – Medication | | | | | | | | |

| **Table S11. Selected Linear SVM discriminative features in EA male sample** | | | | | | |
| --- | --- | --- | --- | --- | --- | --- |
| Weight ranking | Medication | PRS | Functional Connectivity | Marital  Status | Employment status | Betas |
| Positive |  |  |  |  |  |  |
| 1 |  |  | **Lower Gamma**  Rostral middle frontal-lh Inferior temporal-rh |  |  | 0.73 |
| 2 | Other meds |  |  |  |  | 0.50 |
| 3 |  |  | **Lower Theta**  Isthmus cingulate-rh  Rostral middle frontal-lh |  |  | 0.46 |
| 4 |  | EDU_EA_EduYears |  |  |  | 0.41 |
| 5 |  |  | **Lower Theta**  Temporal pole-rh  Transverse temporal-rh |  |  | 0.08 |
| 6 | Sleep meds |  |  |  |  | 0.051 |
| 7 |  | EDU_EA_Neuroticism |  |  |  | 0.045 |
| Negative |  |  |  |  |  |  |
| 1 |  |  | **Higher Gamma**  Superior temporal-lh  lingual-rh |  |  | -0.68 |
| 2 |  | EAGLE_AGGRESSION_EA_AGGRESSION |  |  |  | -0.52 |
| *Note:* lh- Left hemisphere, rh – Right hemisphere, meds – Medication, Lower, Higher coherence of the AUD group compared to the remitted group. Table S15 for PRS description. | | | | | | |

| **Table S12. Selected Linear SVM discriminative features in EA female sample** | | | | | | |
| --- | --- | --- | --- | --- | --- | --- |
| Weight ranking | Medication | PRS | Functional Connectivity | Marital  Status | Employment status | Betas |
| Positive |  |  |  |  |  |  |
| 1 |  |  | **Lower Theta**  Lateral occipital-rh  fusiform-lh |  |  | 0.79 |
| 2 |  |  | **Lower Theta**  paracentral-lh  fusiform-lh |  |  | 0.74 |
| 3 |  |  | **Lower Alpha**  parahippocampal-lh  Frontal pole-lh |  |  | 0.69 |
| 4 |  |  | **Lower Theta**  parahippocampal-lh  Lateral occipital-rh |  |  | 0.641 |
| 5 |  | EDU_EA_EduYears |  |  |  | 0.12 |
| Negative |  |  |  |  |  |  |
| 1 |  |  | **Higher Alpha**  Superior temporal-rh  Inferior parietal-lh |  |  | -0.79 |
| 2 |  |  | **Higher Beta**  Superior parietal-rh  insula-lh |  |  | -0.74 |
| 3 |  | EDU_EA_DS |  |  |  | -0.59 |
| 4 |  |  |  | Marital  Status |  | -0.068 |
| 5 |  | EDU_EA_Neuroticism |  |  |  | -0.021 |
| *Note:* lh- Left hemisphere, rh – Right hemisphere, meds – Medication, Lower, Higher coherence of the AUD group compared to the remitted group. Table S15 for PRS description. | | | | | | |

| **Table S13. Selected Linear SVM discriminative features in AA male sample** | | | | | | |
| --- | --- | --- | --- | --- | --- | --- |
| Weight ranking | Medication | PRS | Functional Connectivity | Marital  Status | Employment status | Betas |
| Positive |  |  |  |  |  |  |
| 1 |  |  | **Lower Gamma**  Medial orbito frontal-rh  Caudal middle frontal-rh |  |  | 1.362 |
| 2 |  |  |  |  | Employment status | 0.52 |
| Negative |  |  |  |  |  |  |
| 1 |  |  | **Higher Theta**  Insula-lh  Inferior parietal-lh |  |  | -1.60 |
| 2 |  |  |  | Marital  Status |  | -1.17 |
| 4 |  | MVP_AA_EA_MAX_ALC_thres20 |  |  |  | -0.24 |
| *Note:* lh- Left hemisphere, rh – Right hemisphere, meds – Medication, Lower, Higher coherence of the AUD group compared to the remitted group. Table S15 for PRS description. | | | | | | |

| **Table S14. Selected Linear SVM discriminative features in AA female sample** | | | | | | |
| --- | --- | --- | --- | --- | --- | --- |
|  |  |  |  |  |  |  |
| Weight ranking | Medication | PRS | Functional Connectivity | Marital  Status | Employment status | Betas |
| **Positive** |  |  |  |  |  |  |
| **1** |  |  | **Lower Theta**  Rostral anterior cingulate-rh  Inferior temporal-lh |  |  | 1.36 |
| 2 |  |  | **Lower Gamma**  precuneus-lh  Posterior Cingulate-lh |  |  | 1.28 |
| 3 |  |  | **Lower Theta**  fusiform-lh  frontal pole-lh |  |  | 1.07 |
| 5 | Depression medication |  |  |  |  | 1.27 |
| **Negative** |  |  |  |  |  |  |
| 1 |  |  | **Higher Beta**  Temporal pole-lh  parsorbitalis-lh |  |  | -1.35 |
| 2 |  |  | **Higer Alpha**  supramarginal-rh  parsorbitalis-rh |  |  | -1.15 |
| **3** |  | EDU_AA_Neuroticism |  |  |  | -0.33 |
| *Note:* lh- Left hemisphere, rh – Right hemisphere, meds – Medication, Lower, Higher coherence of the AUD group compared to the remitted group. Table S15 for PRS description. | | | | | | |

| **Table S15. PRS description** | | |
| --- | --- | --- |
| **PRS** | **GWAS Discovery Sample** | **Description** |
| EDU_EA_EduYears | EDU | Educational attainment, EA |
| EDU_EA_DS | EDU | Depression, EA |
| EDU_EA_Neuroticism | EDU | Neuroticism, EA |
| EDU_AA_SWB | EDU | Subjective wellbeing, EA |
| MVP_AA_EA_MAX_ALC_thres20 | MVP | Million Veteran Program (MVP), AA, EA, is the highest number of drinks a subject reported drinking during a single day in a typical month |
| EAGLE_AGGRESSION_EA_AGGRESSION | EAGLE | Aggression, EA |
| *Note:* EA -European ancestry, AA - African ancestry | | |

**References**

1 Begleiter H, Porjesz B, Reich T, Edenberg HJ, Goate A, Blangero J *et al.* Quantitative trait loci analysis of human event-related brain potentials: P3 voltage. *Electroencephalogr Clin Neurophysiol - Evoked Potentials* 1998. doi:10.1016/S0168-5597(98)00002-1.

2 Edenberg HJ, Bierut LJ, Boyce P, Cao M, Cawley S, Chiles R *et al.* Description of the data from the Collaborative Study on the Genetics of Alcoholism (COGA) and single-nucleotide polymorphism genotyping for Genetic Analysis Workshop 14. *BMC Genet* 2005; **6 Suppl 1**: S2–S2.

3 Reich T. A Genomic Survey of Alcohol Dependence and Related Phenotypes: Results from the Collaborative Study on the Genetics of Alcoholism (COGA). *Alcohol Clin Exp Res* 1996; **20**: 133a–137a.

4 Begleiter H. The Collaborative Study on the Genetics of Alcoholism. *Alcohol Health Res World* 1995; **19**: 228–236.

5 Zheng X, Levine D, Shen J, Gogarten SM, Laurie C, Weir BS. A high-performance computing toolset for relatedness and principal component analysis of SNP data. *Bioinformatics* 2012. doi:10.1093/bioinformatics/bts606.

6 Klem G, Luders H, Jasper H, Elger C. The ten-twenty electrode system of the International Federation. *Electroencephalogr Clin Neurophysiol* 1958; **10**: 371–375.

7 Oostenveld R, Fries P, Maris E, Schoffelen JM. FieldTrip: Open source software for advanced analysis of MEG, EEG, and invasive electrophysiological data. *Comput Intell Neurosci* 2011; **2011**. doi:10.1155/2011/156869.

8 Lopez-Calderon J, Luck SJ. ERPLAB: an open-source toolbox for the analysis of event-related potentials. *Front Hum Neurosci* 2014; **8**. doi:10.3389/fnhum.2014.00213.

9 Gramfort A, Luessi M, Larson E, Engemann DA, Strohmeier D, Brodbeck C *et al.* MEG and EEG data analysis with MNE-Python. *Front Neurosci* 2013. doi:10.3389/fnins.2013.00267.

10 Gramfort A, Luessi M, Larson E, Engemann DA, Strohmeier D, Brodbeck C *et al.* MNE software for processing MEG and EEG data. *Neuroimage* 2014. doi:10.1016/j.neuroimage.2013.10.027.

11 Jas M, Larson E, Engemann DA, Leppäkangas J, Taulu S, Hämäläinen M *et al.* A reproducible MEG/EEG group study with the MNE software: Recommendations, quality assessments, and good practices. *Front Neurosci* 2018. doi:10.3389/fnins.2018.00530.

12 Desikan RS, Ségonne F, Fischl B, Quinn BT, Dickerson BC, Blacker D *et al.* An automated labeling system for subdividing the human cerebral cortex on MRI scans into gyral based regions of interest. *Neuroimage* 2006. doi:10.1016/j.neuroimage.2006.01.021.

13 Dale AM, Liu AK, Fischl BR, Buckner RL, Belliveau JW, Lewine JD *et al.* Dynamic statistical parametric mapping: Combining fMRI and MEG for high-resolution imaging of cortical activity. *Neuron* 2000. doi:10.1016/S0896-6273(00)81138-1.

14 Lin FH, Witzel T, Ahlfors SP, Stufflebeam SM, Belliveau JW, Hämäläinen MS. Assessing and improving the spatial accuracy in MEG source localization by depth-weighted minimum-norm estimates. *Neuroimage* 2006. doi:10.1016/j.neuroimage.2005.11.054.

15 Tibshirani R. Regression Shrinkage and Selection Via the Lasso. *J R Stat Soc Ser B* 2018. doi:10.1111/j.2517-6161.1996.tb02080.x.

16 Knight K, Fu W. Asymptotics for Lasso-type estimators. *Ann Stat* 2000; **28**: 1356–1378.

17 Ghosh D, Chinnaiyan AM. Classification and selection of biomarkers in genomic data using LASSO. *J Biomed Biotechnol* 2005; **2005**: 147–154.

18 Whelan R, Watts R, Orr CA, Althoff RR, Artiges E, Banaschewski T *et al.* Neuropsychosocial profiles of current and future adolescent alcohol misusers. *Nature* 2014. doi:10.1038/nature13402.

19 Kranzler HR, Zhou H, Kember RL, Vickers Smith R, Justice AC, Damrauer S *et al.* Genome-wide association study of alcohol consumption and use disorder in 274,424 individuals from multiple populations. *Nat Commun* 2019. doi:10.1038/s41467-019-09480-8.

20 Okbay A, Beauchamp JP, Fontana MA, Lee JJ, Pers TH, Rietveld CA *et al.* Genome-wide association study identifies 74 loci associated with educational attainment. *Nature* 2016. doi:10.1038/nature17671.

21 Lee JJ, Wedow R, Okbay A, Kong E, Maghzian O, Zacher M *et al.* Gene discovery and polygenic prediction from a genome-wide association study of educational attainment in 1.1 million individuals. *Nat Genet* 2018. doi:10.1038/s41588-018-0147-3.

22 Otowa T, Hek K, Lee M, Byrne EM, Mirza SS, Nivard MG *et al.* Meta-analysis of genome-wide association studies of anxiety disorders. *Mol Psychiatry* 2016. doi:10.1038/mp.2015.197.

23 Schumann G, Liu C, O’Reilly P, Gao H, Song P, Xu B *et al.* KLB is associated with alcohol drinking, and its gene product β-Klotho is necessary for FGF21 regulation of alcohol preference. *Proc Natl Acad Sci U S A* 2016. doi:10.1073/pnas.1611243113.

24 Pappa I, St Pourcain B, Benke K, Cavadino A, Hakulinen C, Nivard MG *et al.* A genome-wide approach to children’s aggressive behavior: The EAGLE consortium. *Am J Med Genet Part B Neuropsychiatr Genet* 2016. doi:10.1002/ajmg.b.32333.

25 Hibar DP, Stein JL, Renteria ME, Arias-Vasquez A, Desrivières S, Jahanshad N *et al.* Common genetic variants influence human subcortical brain structures. *Nature* 2015. doi:10.1038/nature14101.

26 Keers R, Coleman JRI, Lester KJ, Roberts S, Breen G, Thastum M *et al.* A Genome-Wide Test of the Differential Susceptibility Hypothesis Reveals a Genetic Predictor of Differential Response to Psychological Treatments for Child Anxiety Disorders. *Psychother Psychosom* 2016. doi:10.1159/000444023.

27 Gelernter J, Kranzler HR, Sherva R, Almasy L, Koesterer R, Smith AH *et al.* Genome-wide association study of alcohol dependence:significant findings in African- and European-Americans including novel risk loci. *Mol Psychiatry* 2014. doi:10.1038/mp.2013.145.

28 Liu M, Jiang Y, Wedow R, Li Y, Brazel DM, Chen F *et al.* Association studies of up to 1.2 million individuals yield new insights into the genetic etiology of tobacco and alcohol use. Nat. Genet. 2019. doi:10.1038/s41588-018-0307-5.

29 Clarke T-K, Adams MJ, Davies G, Howard DM, Hall LS, Padmanabhan S *et al.* Genome-wide association study of alcohol consumption and genetic overlap with other health-related traits in UK Biobank (N=112 117). *Mol Psychiatry* 2017. doi:10.1038/mp.2017.153.
